# A Review of *N*-(1,3-Dimethylbutyl)-*N*′-phenyl-*p*-Phenylenediamine (6PPD) and Its Derivative 6PPD-Quinone in the Environment

**DOI:** 10.3390/toxics12060394

**Published:** 2024-05-28

**Authors:** Yi Li, Jingjing Zeng, Yongjin Liang, Yanlong Zhao, Shujun Zhang, Zhongyan Chen, Jiawen Zhang, Xingze Shen, Jiabin Wang, Ying Zhang, Yuxin Sun

**Affiliations:** 1 Eco-Environmental Monitoring and Research Center, Pearl River Valley and South China Sea Ecology and Environment Administration, Ministry of Ecology and Environment, Guangzhou 510611, China; liyi@zjnhjg.mee.gov.cn (Y.L.); liangyongjin@zjnhjg.mee.gov.cn (Y.L.); zhaoyanlong@zjnhjg.mee.gov.cn (Y.Z.); ce03ying@zjnhjg.mee.gov.cn (Y.Z.); 2 Guangdong Provincial Key Laboratory of Chemical Pollution and Environmental Safety & MOE Key Laboratory of Environmental Theoretical Chemistry, School of Environment, South China Normal University, Guangzhou 510006, China; zengjingjing22@mails.ucas.ac.cn (J.Z.); 2023024817@m.scnu.edu.cn (S.Z.); 2023024717@m.scnu.edu.cn (Z.C.); 2023024815@m.scnu.edu.cn (J.Z.); 2023024671@m.scnu.edu.cn (X.S.); 2023024799@m.scnu.edu.cn (J.W.); 3 Key Laboratory of Tropical Marine Bio-Resources and Ecology, South China Sea Institute of Oceanology, Chinese Academy of Sciences, Guangzhou 510301, China; 4 University of Chinese Academy of Sciences, Beijing 100049, China

**Keywords:** 6PPD, 6PPD-quinone, environmental distribution, toxicity

## Abstract

As an antioxidant and antiozonant, *N*-(1,3-Dimethylbutyl)-*N*′-phenyl-*p*-phenylenediamine (6PPD) is predominantly used in the rubber industry to prevent degradation. However, 6PPD can be ozonated to generate a highly toxic transformation product called *N*-(1,3-Dimethylbutyl)-*N*′-phenyl-*p*-phenylenediamine quinone (6PPD-quinone), which is toxic to aquatic and terrestrial organisms. Thus, 6PPD and 6PPD-quinone, two emerging contaminants, have attracted extensive attention recently. This review discussed the levels and distribution of 6PPD and 6PPD-quinone in the environment and investigated their toxic effects on a series of organisms. 6PPD and 6PPD-quinone have been widely found in air, water, and dust, while data on soil, sediment, and biota are scarce. 6PPD-quinone can cause teratogenic, developmental, reproductive, neuronal, and genetic toxicity for organisms, at environmentally relevant concentrations. Future research should pay more attention to the bioaccumulation, biomagnification, transformation, and toxic mechanisms of 6PPD and 6PPD-quinone.

## 1. Introduction

*N*-(1,3-Dimethylbutyl)-*N*′-phenyl-*p*-phenylenediamine (6PPD) is an antiozonant and antioxidant which is predominantly used as an additive in synthetic rubber industries, especially in tire products. 6PPD is mainly added to tire preparation formulations at a mass ratio of 0.4–2% to prevent the bending cracking, thermal degradation, and ozone cracking of the rubber materials [1]. It is estimated that approximately 3.1 billion tires are produced every year all over the world [2]. Apart from the direct emissions from rubber-related products, 6PPD in tire and road wear particles (TRWPs) can also be released into the environment and generate new degradation and/or transformation products during the use of vehicles when accelerating, braking, and turning [3]. These potential toxic substances were widely distributed to various environmental media through surface runoff, atmospheric transportation, soil infiltration, and sediment deposition [4]. In addition to various rubber products, 6PPD is also used in the preparation of clothes, hair dye, nail polish dyes, lubricants, and other items, which are closely related to humans’ daily life [5,6]. A recent study showed that 6PPD-quinone, the ozone product of 6PPD, is a major contributor to the death of coho salmon (*Oncorhynchus kisutch*) in the Pacific Northwest of the United States [2,7]. 6PPD and 6PPD-quinone (Table 1) can cause adverse effects to human health through these methods of exposure. Therefore, these two emerging compounds have recently attracted extensive attention.

The widespread existence of 6PPD and 6PPD-quinone was found in different environmental media including water [8,9], air [10], dust [11], soil [9], sediment [12], and even in fish samples [13]. In addition, the widespread occurrence of 6PPD and 6PPD-quinone was also observed in the urine of pregnant women, as well as in adults and children. The potential human health risks after being exposed to 6PPD and 6PPD-quinone are raising concern. The toxic effects of 6PPD and 6PPD-quinone to various organisms in the ecosystem have been widely reported in aquatic organisms such as the same genus for coho salmon (*Oncorhynchus mykiss* and *Oncorhynchus tshawytscha*) [14,15,16], vertebrate embryos [17], mammals [18], single-celled animals [19], mussels [20], crustaceans [21], microorganism [22], and plants [23]. In order to better comprehend the environmental distribution, toxicity, and health risks of 6PPD and 6PPD-quinone, a systematic review about these two emerging compounds is urgently needed. In this review, we summarize the environmental occurrence and toxicity of 6PPD and 6PPD-quinone, as well as highlighting the limitations of existing studies and providing suggestions for future research.

**Table 1:** Basic information about 6PPD and 6PPD-quinone.

Compounds	Abbreviation	CAS No.	Chemical Formula	Molecular Weight	Log*K*_ow_	Structure Diagram
*N*-(1,3-Dimethylbutyl)-*N*′-phenyl-*p*-phenylenediamine	6PPD	793-24-8	C_18_H_24_N_2_	268.40	4.47	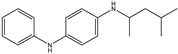
*N*-(1,3-Dimethylbutyl)-*N*′-phenyl-*p*-phenylenediamine quinone	6PPD-quinone	2754428-18-5	C_18_H_22_N_2_O_2_	298.39	3.98	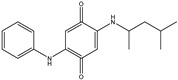

Data are from [12].

## 2. Environmental Occurrence and Fate

### 2.1. Air

**Figure 1:**
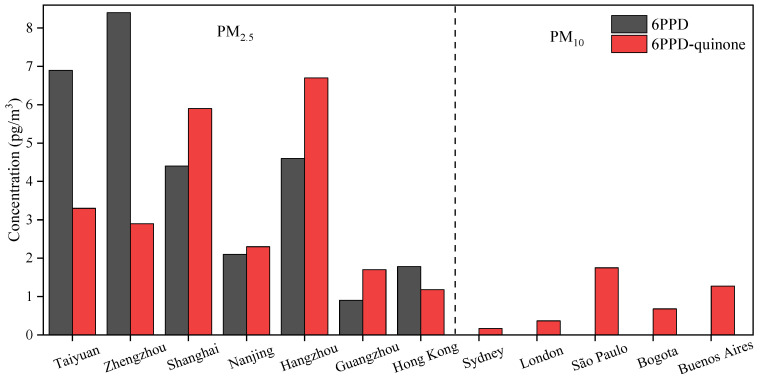
Concentrations of 6PPD and 6PPD-quinone in PM_2.5_ and PM_10_. Data are from [9,24,26].

The smaller-size particles in TRWPs can be transported by air, so 6PPD and 6PPD-quinone widely exist in the air, especially in the micro-environments associated with vehicles. About 3–7% of PM_2.5_ particles are thought to be caused from tire wear. Air intake is an important pathway for exposure to 6PPD and 6PPD-quinone. 6PPD and 6PPD-quinone were widely measured in PM_2.5_ of Chinese cities, with concentrations ranging from 1.02 to 9340 and 2.44 to 7250 pg/m^3^, respectively. 6PPD and 6PPD-quinone were first reported in 81% PM_2.5_ of six typical large cities in Southern and Northern China [24]. The median concentrations of 6PPD and 6PPD-quinone in PM_2.5_ of Taiyuan, Zhengzhou, Shanghai, Nanjing, Hangzhou, and Guangzhou were 6.9, 8.4, 4.4, 2.1, 4.6, and 0.9 pg/m^3^ and 3.3, 2.9, 5.9, 2.3, 6.7, and 1.7 pg/m^3^, respectively. The highest levels of 6PPD and 6PPD-quinone among the six cities were observed in Hangzhou and Zhengzhou, respectively (Figure 1). The concentrations of 6PPD-quinone were similar to those in a study by Wang et al. [25], with levels of 11.1, 8.75, 25.5, 13.1, and 5.25 pg/m^3^ in Taiyuan, Zhengzhou, Shanghai, Hangzhou, and Guangzhou, respectively. The levels of 6PPD and 6PPD-quinone in 2017 ranged from 1.02 to 3190, 2.23 to 9340 pg/m^3^, 2.44 to 1780, and 2.96 to 7250 pg/m^3^ in Taiyuan and Guangzhou, respectively [10]. The levels of the two pollutants in Guangzhou (1820 pg/m^3^ and 1100 pg/m^3^) were much higher than those in Taiyuan (81.0 pg/m^3^ and 744 pg/m^3^), which may be related to car ownership. In 2017, the number of motor vehicles in Guangzhou and Taiyuan were 2.34 million and 1.45 million, respectively [10].

6PPD-quinone was measured in PM_10_ in 18 megacities using the Global Atmospheric Passive Sampling Network, with concentrations ranging from not detected (nd) to 1.75 pg/m^3^ [26]. The highest concentration was observed in São Paulo, Brazil (1.75 pg/m^3^), followed by Buenos Aires, Argentina (1.27 pg/m^3^); Bogota, Colombia (0.68 pg/m^3^); London, UK (0.37 pg/m^3^); and Sydney, Australia (0.17 pg/m^3^) (Figure 1). 6PPD-quinone also widely existed in PM_10_ from waste recycling factories in the Huangpu, Panyu, Nansha, and Huadu districts of Guangzhou, China [1]. The 6PPD-quinone concentrations in the work place were much greater than those in other areas. Moreover, 6PPD-quinone concentrations increased with the increase in particle size. Lower 6PPD-quinone levels were detected in samples with a size of 1.1–2.1 μm, which were in the range of 1.04–3.73 pg/m^3^. The 6PPD-quinone concentrations in sample with a size of 9.0–10.0 μm increased to 7.78–23.2 pg/m^3^. These results indicated that 6PPD-quinone was more inclined to accumulate in coarse particles.

### 2.2. Dust

**Figure 2:**
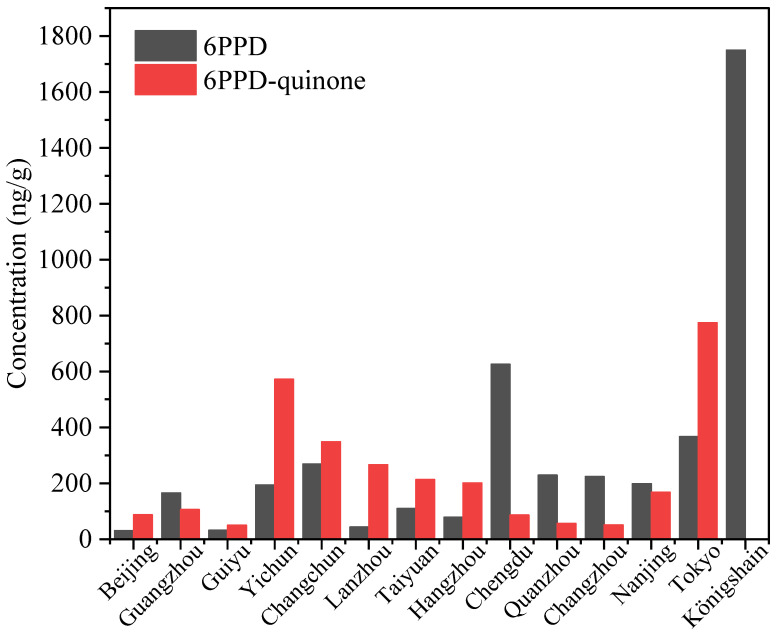
Levels of 6PPD and 6PPD-quinone in dust. Data are from [6,29,30,31,32,33,34,35].

Dust is often regarded as a sink of various contaminants in both indoor and outdoor environments [27,28]. The occurrence of 6PPD and 6PPD-quinone has been found in road dust with concentrations ranging from nd to 1498 and nd to 1327 ng/g, respectively. Generally, the levels of 6PPD and 6PPD-quinone in dust from China were much lower than those from Japan and Germany (Figure 2). The levels of 6PPD in dust from an electronic waste recycling workshop in Yichun (194 ng/g) were much greater than those from other environments, such as vehicles, shopping malls, bedrooms, dormitories, and air-conditioners [5,12,29]. Huang et al. [5] collected dust samples from four types of locations (roads, parking lots, vehicles, and houses) in Guangzhou. The highest median concentrations were found in parking lots (241 ng/g), followed by roads (52.5 ng/g), vehicles (19.3 ng/g), and houses (0.3 ng/g). The concentration of 6PPD in road dust from Guiyu (32.1 ng/g), a traditional e-waste recycling site, was higher than that in the reference area without e-waste pollution (18.5 ng/g) [30]. For 6PPD-quinone, the greatest concentrations were seen in vehicles (80.9 ng/g), parking lots (41.8 ng/g), and roads (32.2 ng/g) [6]. 6PPD and 6PPD-quinone were also measured in dusts from vehicles, female dormitories, male dormitories, residential bedrooms, residential air conditioners, and shopping malls in Guangzhou [5,12,31]. Dust from vehicles had relatively higher levels of 6PPD-quinone (43.0 ng/g), followed by shopping malls (23.5 ng/g), residential air conditioners (11.4 ng/g), residential bedrooms (10.7 ng/g), female dormitories (6.78 ng/g), and male dormitories (4.76 ng/g) [12]. The median concentrations of 6PPD and 6PPD-quinone in road dust (356 and 323 ng/g) were greater than that of parking lots (122 and 154 ng/g) [31]. These results suggested that the levels of 6PPD and 6PPD-quinone in dusts from indoor environments were lower than those from outdoor environments.

6PPD concentrations in dust from the tunnel in Königshain, Germany (1750 ng/g) [32] were much higher than those in arteries and residential road pavements in Tokyo, Japan (329 ng/g) [33], as well as all cities of China (Figure 2). 6PPD in dust from the highway tunnel is difficult to disperse compared with the open road. Relatively high concentrations of 6PPD were detected in dust from Chengdu in China [34], which has the second largest number of cars in China and the third largest number of cars globally (3.89 million). For 6PPD-quinone, the highest concentration was found in the streets of Tokyo, Japan [33], followed by the e-waste recycling plant in Yichun [29]. Among the road dust samples in China, Changchun city had the highest concentration of 6PPD-quinone (349 ng/g), followed by Lanzhou (266 ng/g) and Taiyuan (213 ng/g) [34]. Changchun is famous for its automobile manufacturing industry, with the first automobile manufacturing plant in China and the top-ranking total vehicle output in the country for many years.

### 2.3. Water

The concentrations of 6PPD and 6PPD-quinone are shown in Table S1. The highest levels of 6PPD (210–2710 ng/L) and 6PPD-quinone (210–2430 ng/L) were detected in water from Hong Kong [9]. 6PPD and 6PPD-quinone were also measured in surface runoff from Huizhou and Dongguan cities, with levels ranging from nd to 7.52 ng/L and 0.53 to 1562 ng/L, respectively [36]. The concentrations of 6PPD and 6PPD-quinone in surface runoff in roads were much greater than those in courtyards and farmland. It is noticeable that 6PPD-quinone was found in one water sample in the drinking water source from Guangzhou, with a concentration of 0.25 ng/L. This is the first report of 6PPD-quinone being present in a drinking water source.

**Figure 3:**
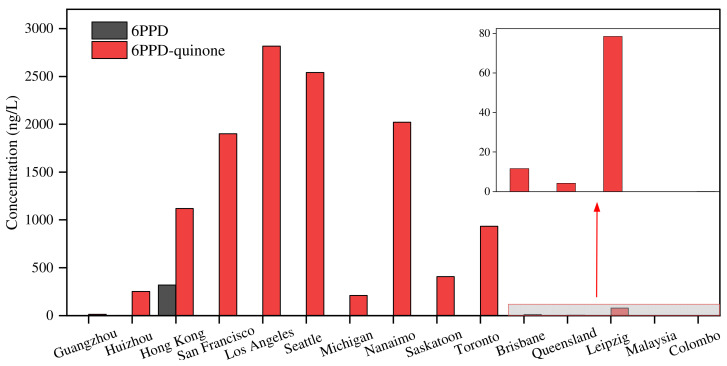
Levels of 6PPD and 6PPD-quinone in water. Data are from [2,8,9,14,36,39,40,41,42,43].

The occurrence of 6PPD and 6PPD-quinone was also reported in water from other countries (Figure 3). The concentrations of 6PPD were generally smaller than the limit of detection. 6PPD-quinone concentrations ranged from nd to 6100 ng/L. The highest concentrations were found in water from Los Angeles (2815 ng/L) [2], followed by Seattle (2540 ng/L), Nanaimo (2022 ng/L) [37], San Francisco (1900 ng/L) [2], Toronto (933 ng/L) [38], and Saskatoon (408.5 ng/L) [39] (Figure 3). Generally, water from road runoff had relatively higher concentrations of 6PPD-quinone. Thus, road runoff can be regarded as an important source of 6PPD-quinone.

6PPD and 6PPD-quinone were measured in the influent and effluent in four typical wastewater treatment plants (WWTPs) in Hong Kong [44]. The concentrations of 6PPD and 6PPD-quinone in the influent of WWTPs were detected to be 1.1–59 and 1.9–470 ng/L. Their concentrations in the effluent of WWTPs decreased to <LOQ-15 and 1.1–37 ng/L, respectively. The median removal efficiency for 6PPD and 6PPD-quinone in WWTPs was 97.5% and 93.6%, indicating that these contaminants can be eliminated in the WWTPs in Hong Kong. 6PPD-quinone was not detected in the influent and effluent of WWTPs in dry weather conditions in Leipzig, Germany [43], but was only found in the influent under snow melting and rainfall conditions, with mean concentrations of 105 and 52 ng/L. The 6PPD-quinone concentrations in the influent during snow melting and rainfall conditions were elevated compared to those in dry weather conditions.

### 2.4. Soil

Few studies have reported on 6PPD and 6PPD-quinone in soil. 6PPD and 6PPD-quinone were measured in all roadside soils from Hong Kong, with levels in the range of 31.4–831 ng/g and 9.50–936 ng/g, respectively [9]. The concentration of 6PPD in soil samples was greater than that of 6PPD-quinone. In soil samples from Guiyu, a traditional e-waste recycling industrial zone, the concentrations of 6PPD and 6PPD-quinone were 0.008–14.6 and 0.002–4.4 ng/g, respectively [30]. The range of concentrations of 6PPD in the surface soil of Harbin’s green-belt soil were 3–233 ng/g, which were lower than those of Hong Kong, but higher than those of Guiyu.

### 2.5. Sediment

The median concentrations of 6PPD and 6PPD-quinone in sediments from urban rivers in the Pearl River Delta, the Pearl River Estuary, and the South China Sea were 14.4 and 9.03, 3.92 and 2.00, and 2.24 and 1.99 ng/g dw, respectively [12]. The concentrations of 6PPD and 6PPD-quinone exhibited a decreasing trend from rivers to open sea. The ratios of 6PPD to 6PPD-quinone also showed a clear decreasing trend. The results suggested that riverine outflows might be an important way to transport 6PPD and 6PPD-quinone to coastal and open sea areas. Additionally, the concentrations of 6PPD and 6PPD-quinone in the sediments from Jiaojiang River were higher, at 25 and 19 ng/g, respectively [45].

### 2.6. Biota

Data on 6PPD and 6PPD-quinone in biota are scarce. 6PPD and 6PPD-quinone were measured in ten fish including freshwater fish and marine fish bought from the aquatic product market in Beijing [13]. 6PPD was detected in the snakehead (0.669 μg/kg) and weever (0.481 μg/kg), while 6PPD-quinone was only detected in the Spanish mackerel, with concentrations lower than the limits of quantification. 6PPD and 6PPD-quinone can cause toxicity to biota. Thus, more attention should be paid to the bioaccumulation of 6PPD and 6PPD-quinone in aquatic products.

### 2.7. Human Urine

6PPD and 6PPD-quinone were first reported in human urine of three population groups including adults (*n* = 50), children (*n* = 50), and pregnant women (*n* = 50) from Guangzhou in South China. The detection frequency of 6PPD and 6PPD-quinone in urine samples were 60% and 100%, respectively. Notably, the median concentration of 6PPD and 6PPD-quinone in the urine of pregnant women (0.068 ng/mL and 2.91 ng/mL) were much higher than those in adults (0.018 ng/mL and 0.40 ng/mL) and children (0.015 ng/mL and 0.076 ng/mL). 6PPD-quinone concentrations in urine were significantly higher than those of 6PPD [46]. The 6PPD levels in adults’ urine samples were lower than those detected in Quzhou (1.1 ng/mL) [47]. It was found that there was a positive relationship between concentrations of 6PPD and 6PPD-quinone in urine, indicating some co-exposure pathways. The pregnant women showed significantly greater 6PPD and 6PPD-quinone concentrations than adults and children, which may be related to a different metabolic rate and/or different exposure sources.

## 3. Toxicity

### 3.1. Salmonid

The acute toxicity of 6PPD or 6PPD-quinone to different species is shown in Table 2. A study published in 2021 [2] first identified and characterized 6PPD-quinone, revealing its acute toxicity to coho salmon (*Oncorhynchus kisutch*, LC_50_ = 0.79 μg/L), a level which was subsequently revised to 95 ng/L [7]. The concentrations of 6PPD-quinone in water were relatively high (<0.3–19 mg/L). Therefore, toxicity tests for 6PPD and 6PPD-quinone have been frequently studied in various fish, especially those in the same genus of salmon family, which is close to *Oncorhynchus kisutch*. The detailed toxic effects of 6PPD and 6PPD-quinone and their related substances to various organisms is shown in Table S2. Juvenile coho exhibited symptoms of gasping, erratic swimming, loss of equilibrium, and increased ventilation after exposure to 6PPD-quinone, with an LC_50_ of 41.0 ng/L, which was lower than the values reported by Tian et al. [7]. The different toxic effects might be ascribed to the differences in size and age of the species. The LC_50_ of 6PPD-quinone for Brook Trout (*Slavelinus fontinslis*) at 24 h was 0.59 μg/L, with behaviors of hovering near the water surface, increased opercular movements, spiraling, and gasping [48]. *Rainbow trout* (*Oncorhynchus mykiss*) showed increased respiration, wheezing, spiraling behavior, loss of balance, and significantly increased blood glucose concentration, with an LC_50_ of 1.96 μg/L at 24 h and 1.00 μg/L at 72 h. Acute toxicity tests for 96 h on three salmon species, including *Salvelinus lencomaenis pluvius*, *Salvelinus curilus*, and *Oncorhynchus masou masou*, showed that 6PPD-quinone was lethal to *Salvelinus leucomaenis pluvius*, with an LC_50_ of 0.51 μg/L at 24 h, and exhibited abnormal swimming behavior, with wandering near the surface, unstable swimming, and rolling phenomena [49]. The results suggested that 6PPD-quinone may share a common mechanism of toxicity to salmonidae.

The enantioselective toxicity of 6PPD and 6PPD-quinone to *Oncorhynchus mykiss* was studied. Mortality was not observed, even at the maximum exposure concentration of 6PPD (400 μg/L) [3]. The LC_50_ values of rac-6PPD-quinone, R-6PPD-quinone, and S-6PPD-quinone at 96 h were 2.26, 4.31, and 1.66 μg/L, respectively. The toxicity of S-6PPD-quinone was greater than that of rac-6PPD-quinone and R-6PPD-quinone. The LC_50_ values of 6PPD for *Oncorhynchus kisutch* and *Oncorhynchus mykiss* were lower than the formation concentrations of S-6PPD-quinone and R-6PPD-quinone in aqueous solution. The species-specific differences in the toxicity of 6PPD-quinone should raise concern.

### 3.2. Zebrafish

Unbalance and chaotic trajectories, anxiety-like behaviors, and hypoactivity were induced for adult zebrafish, after exposure to 6PPD and 6PPD-quinone for 12 h [54]. Behavioral toxicity triggered by 6PPD and 6PPD-quinone was also seen in zebrafish larvae. The LC_50_ values for zebrafish larvae at 24 h were 1384.9 μg/L for 6PPD and 308.6 μg/L for 6PPD-quinone, respectively [52]. High levels of 6PPD and 6PPD-quinone interfered with the typical development of zebrafish larvae. Specifically, the hatching rate, autonomic motor ability, swimming performance, and heartbeat per minute of zebrafish larvae decreased, their body length shortened, and their oxygen consumption increased [17,52]. The growth and development of zebrafish larvae/embryos was influenced after their exposure to 6PPD. 6PPD can also cause oxidative stress for zebrafish juveniles [17]. The freshwater zebrafish exhibited a similar toxic mechanism to other zebrafish [21]. The effect of hatching rates, body length, and the curvature of the spine were observed. In addition to a series of toxicity symptoms, 6PPD can also bioaccumulate in zebrafish with a concentration of 2658 ng/g wet weight, after exposure to 100 ng/g of 6PPD in water for 12 h [53].

### 3.3. Other Species

A kind of freshwater fish (*Oryzias latipes*) showed fatal sensitivity to 6PPD, with a mortality rate of 80% within 96 h and abnormal swimming behavior within 1 h, while 6PPD-quinone did not show lethal toxicity or abnormal behavioral symptoms [21]. Similarly, 6PPD caused damage to two crustacean species (*Hyalella azteca* and *Daphnia magna*), with a mortality rate of 100% at 138 μg/L and 286 μg/L, respectively [21]. The toxicity of 6PPD-quinone to *Hyalella azteca* and *Daphnia magna* was not observed. In addition to the acute toxicity test, the long-term toxicity of 6PPD has also been studied. The population growth and reproductive rate was affected for *Brachionus calyciflorus* after exposure to 6PPD for 12 days [4]. A recent exposure experiment of 4.5 days on a species of nematodes, *Caenorhabditis elegans*, 100 μg/L group could cause lethality and all exposure concentrations lead to varying degrees of intestinal toxicity [55]. Hypoactivity, diarrhea, bradypnea, hypothermia, and prone position were observed for rats after exposure to 6PPD and 6PPD-quinone. Liver weight increased significantly after the exposure of male mice to them.

### 3.4. In Vitro Experiment Level

In vitro experiments are helpful to understand the toxic mechanism of pollutants. 6PPD inhibits cell proliferation and promotes apoptosis for human cells [7]. The proliferation inhibition rate of human embryonic lung fibroblasts (HELF) cells was enhanced with increasing levels of 6PPD concentration [7]. The apoptosis rate will increase with increasing levels of 6PPD concentration. 6PPD could inhibit the growth of HELF cells and induce cell apoptosis. 6PPD-quinone and deoxyguanosine would produce the conjugate 6PPD-quinone-dG, and 6PPD-quinone treated amounts were positively correlated with 6PPD-quinone-dG production, which is negatively correlated with cell viability, resulting in sublethal effects on single-celled algae [56].

## 4. Summary and Perspectives

The occurrence of 6PPD and 6PPD-quinone are detected in the atmosphere, water, soil, dust, sediments, and organisms. The concentration of 6PPD-quinone is generally increased during rainstorm events. 6PPD-quinone is more stable than 6PPD. 6PPD and 6PPD-quinone have toxicity effects, especially to aquatic species of salmon. Different species have a distinct sensitivity to these two chemicals; their toxic mechanisms need to be further studied. It is suggested that future research can be focused on the following aspects:

1. 6PPD and 6PPD-quinone are lipophilic compounds, but the bioavailability of 6PPD-quinone remains unknown. Therefore, it should be considered to focus on bioaccumulation, trophic magnification, and biotransformation in aquatic/terrestrial biota.
2. Although the acute toxicity of 6PPD and 6PPD-quinone to different organisms has been studied, the long-term toxicity of these two contaminants to biota are far from sufficient. The mechanism of toxicity on 6PPD and 6PPD-quinone needs to be explored.
3. Both 6PPD and 6PPD-quinone are chiral compounds. In future research, attention should be paid to the environmental behaviors and toxicity of their enantiomers.
4. Little information is available about human exposure to 6PPD and 6PPD-quinone. Future studies need to focus on their exposure to humans.

## Figures and Tables

**Table 2:** Acute toxicity of 6PPD and 6PPD-quinone.

Chemical	Species	LC_50_	Reference
6PPD-quinone	*Oncorhynchus kisutch* (>1 year)	95 ng/L (24 h)	[2]
6PPD-quinone	*Oncorhynchus kisutch* (~3 weeks)	41.0 ng/L (24 h)	[15]
6PPD-quinone	*Oncorhynchus kisutch* (189 days)	80.4 ng/L (24 h)	[50]
6PPD-quinone	*Oncorhynchus mykiss* (~2 years)	1.96 μg/L (24 h) 1.00 μg/L (72 h)	[48]
6PPD-quinone	*Oncorhynchus mykiss* (3 months)	0.90 μg/L (24 h)	[51]
6PPD-quinone	*Slavelinus fontinslis* (~1 year)	0.59 μg/L (24 h)	[48]
6PPD-quinone	*Salvelinus lencomaenis pluvius* (<1 year)	0.51 μg/L (24 h)	[49]
rac-6PPD-quinone	*Oncorhynchus mykiss*	2.26 μg/L (96 h)	[3]
R-6PPD-quinone	*Oncorhynchus mykiss*	4.31 μg/L (96 h)	
S-6PPD-quinone	*Oncorhynchus mykiss*	1.66 μg/L (96 h)	
6PPD-quinone	zebrafish larvae (116 hpf)	308.7 μg/L (24 h) 132.9 μg/L (96 h)	[52]
6PPD-quinone	zebrafish embryos/larvae (2 hpf)	2200 μg/L (96 h)	[17]
rac-6PPD	*Gobiocypris rarus*	162 μg/L (96 h)	[3]
R-6PPD	*Gobiocypris rarus*	201 μg/L (96 h)	
S-6PPD	*Gobiocypris rarus*	201 μg/L (96 h)	
6PPD	zebrafish larvae (116 hpf)	1384.9 μg/L (24 h)	[52]
6PPD	zebrafish larvae (2–96 hpf)	737 μg/L (12 h)	[53]

## Data Availability

Data will be made available upon reasonable request.

## Supplementary Materials

The following supporting information can be downloaded at: https://www.mdpi.com/article/10.3390/toxics12060394/s1, Table S1: Occurrence of 6PPD and 6PPD-quinone in various environmental media; Table S2: Biological toxicity of 6PPD and 6PPD-quinone. References [57,58,59,60,61,62,63,64,65,66,67] were cited in the Supplementary Materials.
