# Supplementary material for: A Review of N-(1,3-Dimethylbutyl)-N′-phenyl-p-Phenylenediamine (6PPD) and Its Derivative 6PPD-Quinone in the Environment
Source: Toxics. 2024 May 28;12(6):394. doi: 10.3390/toxics12060394 (PMC11209267; doi:10.3390/toxics12060394)
Supplement: Supplementary file 1 [file toxics-12-00394-s001.zip › toxics-2983363-supplementary.pdf]

Supporting Information

A Review of *N*-(1,3-Dimethylbutyl)-*N′*-phenyl-*p*-Phenylenediamine (6PPD) and Its Derivative 6PPD-Quinone in the Environment

Yi Li[1,†], Jingjing Zeng[2,3,4,†], Yongjin Liang[1], Yanlong Zhao[1], Shujun Zhang[2], Zhongyan Chen[2], Jiawen Zhang[2], Xingze Shen[2], Jiabin Wang[2], Ying Zhang[1] and Yuxin Sun[2,*]

[1] Eco-Environmental Monitoring and Research Center, Pearl River Valley and South China Sea Ecology and Environment Administration, Ministry of Ecology and Environment, Guangzhou 510611, China; liyi@zjnhjg.mee.gov.cn(Y.L.); liangyongjin@zjnhjg.mee.gov.cn(Y.L.); zhaoyanlong@zjnhjg.mee.gov.cn (Y.Z.); ce03ying@zjnhjg.mee.gov.cn(Y.Z.)

[2] Guangdong Provincial Key Laboratory of Chemical Pollution and Environmental Safety & MOE Key Laboratory of Environmental Theoretical Chemistry, School of Environment, South China Normal University, Guangzhou 510006, China; zengjingjing22@mails.ucas.ac.cn(J.Z.); 2023024817@m.scnu.edu.cn (S.Z.); 2023024717@m.scnu.edu.cn(Z.C.); 2023024815@m.scnu.edu.cn (J.Z.); 2023024671@m.scnu.edu.cn(X.S.); 2023024799@m.scnu.edu.cn (J.W.)

[3] Key Laboratory of Tropical Marine Bio-resources and Ecology, South China Sea Institute of Oceanology, Chinese Academy of Sciences, Guangzhou 510301, China;

[4] University of Chinese Academy of Sciences, Beijing 100049, China;

† These authors contributed equally to this study.

* Correspondence:sunyuxin@m.scnu.edu.cn (Y.S.)

Table S1. Occurrence of 6PPD and 6PPD-quinone in various environmental media.

Environmental media	Area, country	Sampling time	Sampling location	Number of samples	6PPD-quinone	6PPD	Reference
	Guangzhou	May 2017 ~ April 2018	Guangdong University of Technology	24	1100 pg/m^3(a)	1820 pg/m^3(a)	[1]
	Guangzhou		roadside	24	2810 pg/m^3(a)	4040 pg/m^3(a)	
	Taiyuan		Shanxi University	24	744 pg/m^3(a)	81.0 pg/m^3(a)	
	Guangzhou	2018 ~ 2021	Guangdong University of Technology	22	5.25 pg/m^3(a)		[2]
	Hangzhou		Binjiang District	7	13.1 pg/m^3(a)		
	Shanghai		Fudan University	4	25.5 pg/m^3(a)		
	Taiyuan		Shanxi University	14	11.1 pg/m^3(a)		
$PM_{2.5}$	Zhengzhou		Zhengzhou University	14	8.75 pg/m^3(a)		
	Taiyuan	2018 ~ 2019	Shanxi University	24	3.3 pg/m^3(a)	6.9 pg/m^3(a)	[3]
	Zhengzhou		Zhengzhou University	12	2.9 pg/m^3(a)	8.4 pg/m^3(a)	
	Shanghai		Fudan University	8	5.9 pg/m^3(a)	4.4 pg/m^3(a)	
	Nanjing		Centers for Disease Control and Prevention	6	2.3 pg/m^3(a)	2.1 pg/m^3(a)	
	Hangzhou		Hangzhou Binjiang	7	6.7 pg/m^3(a)	4.6 pg/m^3(a)	
	Guangzhou		Guangdong University of Technology	24	1.7 pg/m^3(a)	0.9 pg/m^3(a)	
	Hong Kong	September 2020 ~ August 2021	Baptist University	16	1.18 pg/m^3(a)	1.78 pg/m^3(a)	[4]
PM_{10}	Huangpu, Guangzhou	September ~	subareas in waste recycling plants	6	40.0 ng/g(b)		[5]

	Panyu, Guangzhou	December 2020	(settled dust-bound)	6	60.7 ng/g(b)		
	Huadu, Guangzhou			6	24.0 ng/g(b)		
	Nansha, Guangzhou			6	186 ng/g(b)		
	Huangpu, Guangzhou	September ~	waste recycling plants	5	0.34 μg/g(b)		
	Panyu, Guangzhou	December 2020		5	0.26 μg/g(b)		
	Huadu, Guangzhou			5	0.21 μg/g(b)		
	Nansha, Guangzhou			5	0.37 μg/g(b)		
	18 major cities in the world	2018-2019	areas away from significant motorways	18	0.847 pg/m^3(b)	ND/<LOQ	[6]
	Sydney, Australia	April ~ July 2018		1	0.170 pg/m^3		
	London, UK	April ~ July 2018		1	0.367 pg/m^3		
	São Paulo, Brazil	April ~ July 2018		1	1.75 pg/m^3		
	Bogota, Colombia	April ~ July 2018		1	0.680 pg/m^3		
	Buenos Aires, Argentina	February ~ May 2019		1	1.27 pg/m^3		
Dust	Guangzhou	2020	road	20	32.2 ng/g(a)	52.5 ng/g(a)	[7]
			vehicle	11	80.9 ng/g(a)	19.3 ng/g(a)	
			mall parking lot	10	41.8 ng/g(a)	241 ng/g(a)	
			house	18	<LOQ	0.3 ng/g(a)	
	Guangzhou	January 2021	road	10	122 ng/g(a)	356 ng/g(a)	[8]
			indoor parking lot	10	154 ng/g(a)	323 ng/g(a)	
	Guangzhou	April ~ August 2021	vehicle	17	43 ng/g(b)		[5]
			female dormitory	16	6.78 ng/g(b)		
			male dormitory	16	4.76 ng/g(b)		
			residential bedroom	16	10.7 ng/g(b)		

		residential air conditioner	18	11.4 ng/g(b)		
		shopping mall	20	23.5 ng/g(b)		
		total	103	17.1 ng/g(b)		
Yichun	August 2020	E-waste recycling workshops	45	375 ng/g(a);<br>572 ng/g(b)	113 ng/g(a);<br>194 ng/g(b)	[9]
Guiyu		road dust	27	17.4346 ng/g(a)		[10]
		house dust	74	3.1959 ng/g(a)		
		kindergarten dust	47	7.5203 ng/g(a)		
Haojiang		road dust	13	30.2690 ng/g(a)		
		house dust	17	1.3528 ng/g(a)		
		kindergarten dust	5	1.3465 ng/g(a)		
Guiyu	March 2012 ~ May	exposed group	11	50.054 ng/g	32.107 ng/g	[11]
Haojiang	2013	reference group	5	105.152 ng/g	18.498 ng/g	
Guiyu	November 2020	exposed group	11	15.400 ng/g		
Haojiang		reference group	6	59.096 ng/g		
Beijing	2019	indoor living room	30		16.4 ng/g (c)	[12]
		outdoor rubber playground	30		30.4 ng/g (c)	
Dalian	September 2022 ~	urban trunk dust	7	47.35 ng/g(a)	3.52 ng/g(a)	[13]
	March 2023	residential dust	7	10.07 ng/g(a)	1.95 ng/g(a)	
		residential plaza dust	5	5.855 ng/g(a)	1.485 ng/g(a)	
Harbin	October 2021 and April 2022	road dust	228		114 ng/g(a)	[14]
	October 2021 and April 2022	garage dust	24		177 ng/g(a)	
	April 2022	parkinglot dust	45		168 ng/g(a)	

	Hangzhou	July 2022 ~ August 2022	indoor dust	97	9.5 ng/g(a); 14 ng/g(b)	10 ng/g(a); 17 ng/g(b)	[15]
	55 cities of China	August 2021 ~ December 2022	expressway service area road dust	5	107.8 ng/g(a)	39.2 ng/g(a)	[16]
			suburban road dust	5	9.6 ng/g(a)	5.6 ng/g(a)	
			urban tunnel road dust	14	171.9 ng/g(a)	100.3 ng/g(a)	
			urban trunk road dust	23	60.6 ng/g(a)	13.1 ng/g(a)	
	Tokyo, Japan	May ~ October 2021	artery and residential road pavement	22	809 ng/g(a)	329 ng/g(a)	[17]
	Königshain, Germany	January 2020	middle of the freeway tunnel	3	220 peak area/mg(b)	1.5 ng/g(b)	[18]
			end of highway tunnel	3	270 peak area/mg(b)	1.9 ng/g(b)	
Water	San Francisco, USA	2019	urban runoff	10	1.9 μg/L(a)		[19]
	Los Angeles, USA	2018 ~ 2019	roadway runoff	2	5.1 μg/L(a)		
			urban runoff from rainstorm events	7	0.53 μg/L(a)		
	Seattle, USA	2018	roadway runoff	16	6.1 μg/L(a)		
	Seattle, USA	October 2018	urban roadway runoff from storm events		0.59 μg/L		[20]
	Hong Kong	August 2021	urban runoff (heavy traffic)	9	1.12 μg/L(a)	0.32 μg/L(a)	[4]
	Hong Kong	October ~ November 2021	wastewater (WWTPs influent)	40	53 ng/L(a)	12 ng/L(a)	[21]
			wastewater (WWTPs effluent)		3.4 ng/L(a)	0.30 ng/L(a)	
	Saskatoon, Canada	June ~ August 2019	rain water outlet	21	593 ng/L(b)		[22]
		March ~ May 2019	snowmelt	10	367 ng/L(b)		
		2020	snowmelt	22	81 ng/L(b); 15-172 ng/L		
	Toronto, Canada	August 2020	wastewater treatment plant (WWTP) outlet	3	0.05 μg/L(b)	<LOD	[23]

		upstream of WWTP	3	<LOD	<LOD	
		downstream of WWTP	3	<LOD	<LOD	
	July 2020	Don River (storm event)	3	0.54 µg/L(b)	<LOD	
		Highland Creek (storm event)	3	0.72 µg/ L(b)	<LOD	
	August 2020	Don River (storm event)	3	0.11 µg/ L(b)	ND	
		Highland Creek (storm event)	3	0.21 µg/ L(b)	ND	
	October 2019 ~	Don River (storm event)	28	2.1 µg/L(a)		[24]
	March 2020	Don River (storm event/ snowmelt)	42	0.6 µg/L(a)		
	October 2019	Don River (storm event)	3	1.91 µg/L(b)		
	October 2019	Don River (storm event)	3	2.30 µg/L(b)		
	January 2020	Don River (storm event)	3	0.60 µg/L(b)		
	March 2020	Don River (snowmelt)	3	0.87 µg/L(b)		
	March 2020	Don River (storm event)	3	0.30 µg/L(b)		
Brisbane, Australia	June 2020	tributary of the Brisbane River	4	5.75 ng/L(a); 17.5 ng/L(b)		[25]
Brisbane, Australia	October 2020	tributary of the Brisbane River (storm event)	28	19 ng/L(a); 21.2 ng/L(b)		
4 regional urban centers in Queensland, Australia	May ~ July 2021	surface water (storm event)	3	7.5 ng/L(a); 11.7 ng/L(b)		[26]
		surface water	6	1.01 ng/L(a); 1.24 ng/L(b)		
		surface water	5	0.12 ng/L(a); 0.13 ng/L(b)		
		surface water	4	1.63 ng/L(a); 2.16 ng/L(b)		

Brisbane, Australia		surface water	3	3.1 ng/L(a); 4.5 ng/L(b)		
Nanaimo, Canada	May ~ June 2021	surface water	2	104 ng/L(a); 104 ng/L(b)		[27]
		stormwater	4	3053 ng/L(a); 2934 ng/L(b)		
	2016	used tire fragments suspension	1	4255 ng/L	210 ng/L	
	2014		1	4009 ng/L	397 ng/L	
	2009		1	463 ng/L	70 ng/L	
		artificial turf pellets suspension	1	59 ng/L	BDL	
Leipzig, Germany	February 2021	WWTPs influent (snow melting event)	6	0.105 μg/L(b)	4.4 μg/L(b)*	[28]
	March 2021	WWTPs influent (rain event)	6	0.052 μg/L(b)	14.3 μg/L(b)*	
	June 2021	WWTPs influent (dry weather condition)	10	ND	0.9 μg/L(b)*	
	February 2021	WWTPs effluent (snow melting event)	6	ND	2.4 μg/L(b)*	
	March 2021	WWTPs effluent (rain event)	6	ND	11.2 μg/L(b)*	
	June 2021	WWTPs effluent (dry weather condition)	10	ND	0.3 μg/L(b)*	
Malaysia	June ~ August 2017	municipal WWTPs influent	39	ND		[29]
		hospital WWTPs influent	37	ND		
	August 2017	industrial WWTPs influent	14	ND		
	June ~ August 2017	municipal WWTPs effluent	39	ND		
		hospital WWTPs effluent	37	ND		

	August 2017	industrial WWTPs effluent	14	ND(a);<br>0.016 ng/L (b)		
Colombo, Sri Lanka	March 2018	municipal WWTPs influent	7	ND(a);<br>0.10 ng/L (b)		
Hikkaduwa, Sri Lanka	March 2018	municipal WWTPs influent	7	ND		
Colombo, Sri Lanka	March 2018	municipal WWTPs effluent	7	ND(a);<br>0.052 ng/L (b)		
Hikkaduwa, Sri Lanka	March 2018	municipal WWTPs effluent	7	ND		
Malaysia	June ~ August 2017	WWTPs influent	45	ND		
Sri Lanka	March 2018	WWTPs influent	45	ND(a);<br>0.052 ng/L (b)		
Malaysia	June ~ August 2017	WWTPs effluent	14	ND(a);<br>0.0025 ng/L (b)		
Sri Lanka	March 2018	WWTPs effluent	14	ND(a);<br>0.026 ng/L (b)		
Guangzhou Liuxi River	January 2020	surface water	19	0.18 ng/L(a);<br>0.23 ng/L(b)	ND	[30]
	July 2020	ground water	43	0.11 ng/L(a);<br>0.13 ng/L(b)	ND	
	April 2021	stormwater	10	0.34 ng/L(a);<br>0.46 ng/L(b)	ND	
	2020	suspended particles	10	0.01 ng/L(a);<br>0.02 ng/L(b)	0.20 ng/L(a);<br>0.27 ng/L(b)	
Jiaojiang River	October 2022	surface water	30	6.1 ng/L(a);<br>7.0 ng/L(b)	10 ng/L(a);<br>12 ng/L(b)	[31]

	Huizhou and Dongguan	June ~ December 2015	courtyard	21	51.6 ng/L(a); 122 ng/L(b)	0.89 ng/L(a); 0.72 ng/L(b)	[32]
			road		576 ng/L(a); 636 ng/L(b)	3.05 ng/L(a); 3.36 ng/L(b)	
			farmland		0.73 ng/L(a); 1.61 ng/L(b)	ND	
	Guangzhou	April 2016	WWTPs influent	1	69.8 ng/L		
			WWTPs influent	1	14.2 ng/L		
	Guangzhou	April 2016	WWTPs influent	1	69.8 ng/L		
			WWTPs influent	1	14.2 ng/L		
			WWTPs influent	1	69.3 ng/L		
			WWTPs effluent	1	ND		
			WWTPs effluent	1	2.09 ng/L		
			WWTPs effluent	1	ND		
		March and July 2015	Zhujiang River surface water	13	1.51ng/L(a); 2.34 ng/L(b)	0.48ng/L(a); 0.56 ng/L(b)	
			Dongjiang River surface water	13	0.91ng/L(a); 1.69 ng/L(b)	0.36ng/L(a); 0.47 ng/L(b)	
		August 2020	Liuxi River source water	1	0.25 ng/L		
			drinking water treatment plants	6	ND		
Sediment	Pearl River Delta	August 2021	surface sediments (urban rivers)	32	9.03 ng/g(a)	14.4ng/g(a)	[33]
	Pearl River Estuary	March 2021	estuary	21	2.00 ng/g(a)	3.92 ng/g(a)	
	South China Sea	March 2021	coasts	20	1.27 ng/g(a)	1.82 ng/g(a)	
			deep-sea regions	12	2.71 ng/g(a)	2.66 ng/g(a)	
	Jiaojiang River	October 2022	surface sediment	30	19 ng/g(a);	25 ng/g(a);	[31]

					14 ng/g(b)	31 ng/g(b)	
Dust leaching solution	Tokyo, Japan	July 2021	artery road	1	3.2 μg/L(e)		[17]
		September 2021	residential road	1	2.3 μg/L(e)		
Soil	Hong Kong	August ~ September 2021	roadside	12	234 ng/g(a)	309 ng/g(a)	[4]
	Dalian	September 2022 ~ March 2023	agricultural land topsoil	13	5.72 ng/g(a)	2.14 ng/g(a)	[13]
	Harbin	October 2021 and April 2022	green-belt soil	156		24.3 ng/g(a)	[14]
	Guiyu	March 2012 ~ May 2013	exposed group	11	0.169 ng/g	0.737 ng/g	[11]
	Haojiang		reference group	5	0.068 ng/g	0.469 ng/g	
	Guiyu	November 2020	exposed group	11	0.772 ng/g	1.687 ng/g	
Fresh atmospheric snow	Anyang	January ~ February 2019	Anyang Normal University	6	23 pg/g (a)	36 pg/g (a)	[34]
	Xinxiang		Henan Normal University	4	12 pg/g (a)	21 pg/g (a)	
	Zhengzhou		Tianjian Lake Park	9	5.6 pg/g (a)	13 pg/g (a)	
	Luoyang		Henan University of Science and Technology	10	24 pg/g (a)	31 pg/g (a)	
	Pingdingshan		Pingdingshan University	8	104 pg/g (a)	11 pg/g (a)	
	Nanyang		Nanyang Institute of Technology	9	14 pg/g (a)	260 pg/g (a)	
	Xinyang		Xinyang Normal University	3	nd	nd	
Rubber particles	Cities in 14 European countries	June ~ November 2017	sport field uncoated	47		570.53 mg/kg(d)	[35]
			sport field coated	10		65.67 mg/kg(d)	
			sport field non-ELT	10		330.82 mg/kg(d)	
			recycles uncoated	25		1478.6 mg/kg(d)	

			recycles coated	4		119.2 mg/kg(d)	
	Nanaimo, Canada	2016	fragments obtained from tires	1	8.52 μg/g	0.42 μg/g	[27]
		2014		1	7.88 μg/g	0.78 μg/g	
		2009		1	0.87 μg/g	0.13 μg/g	
			artificial turf pellets	1	0.11 μg/g	BDL	
TWPs	Washington, USA		TWPs	1	12 μg/g	2300 μg/g	[36]
Crumb rubber		May-21	crumb rubber	9	9.8 μg/g(a)	1.2 μg/g(a)	
Elastomeric consumer products			doormat	2	18 μg/g(a)	630 μg/g(a)	
			sneaker sole	3	0.48 μg/g(a)	0.14 μg/g(a)	
			laboratory stopper	3	0.47 μg/g(a)	0.71 μg/g(a)	
			garden hose	1	<LOD	0.071 μg/g	
Urine	Guangzhou	April ~June 2022	adults	50	0.40 ng/mL(a)	0.018 ng/mL(a)	[37]
			children	50	0.076 ng/mL(a)	0.015 ng/mL(a)	
			pregnant women	50	2.91 ng/mL(a)	0.068 ng/mL(a)	
	Quzhou	April 2019	adults	151		1.1ng/mL(a); 1.2ng/mL(b)	[38]
Blood	South China	December 2021 ~ June 2022	serum	281	0.15 ng/mL(a); 0.17 ng/mL(b)	0.063 ng/mL(a); 0.082 ng/mL(b)	[39]
White-spotted char *(Salvelinus lencomaenis pluvius)*			brain		4.0 μg/kg		[40]
			gill		6.2 μg/kg		
Southern Asian Dolly Varden *(Salvelinus curilus)*			brain		25 ± 5.2 μg/kg		
			gill		70 ± 65 μg/kg		

Landlocked masu salmon *(Oncorhynchus masou masou)*	brain	4.7 ± 2.6 μg/kg		
	gill	38 ± 15 μg/kg		
Zebrafish larvae		2,657 ng/g		[41]
Lettuce	leave	-2.19μg/g	-0.78 μg/g	[42]
Snakehead fish			0.669 μg/kg	[43]
Weever			0.481 μg/kg	
Spanish mackerel			<LOQ	
Male mice	liver	1194.687±515.861 ng/g liver weight	681.954 ±65.037 ng/g liver weight	[44]

Note: LOQ = Limit of quantification; LOD = Limit of detection; ND = Not Determined; BDL = Below Detection Limit; (a) Median concentration; (b)Mean concentration; (c)Geometric mean concentration; (d) Arithmetic mean concentration; (e) Equilibrium concentration of first order kinetic model; (*) Concentration of 6PPD and TPs.

Table S2. Biological toxicity of 6PPD and 6PPD-quinone.

Species	Poison	Tested species	Treatment	Physiological change	Reference
salmonid	6PPD-quinone	juvenile coho salmon (*Oncorhynchus kisutch*) (1+ years)	~20 μg/L, 24 h	Highly toxic (<5 hours, with initial symptoms evident within 90 min), LC_{50}=0.79±0.16 μg/L.	[20]
	TWP leachate		250 mg/L, 72 h, 80°C	100% mortality with behavior of circling, surface gaping, and equilibrium loss.	
	TWP leachate		250 mg/L, 24 h	Acutely and rapidly lethal (98.5% mortality）, LC_{50}=0.82±0.27 μg/L.	
	6PPD-quinone	juvenile coho salmon (*Oncorhynchus kisutch*)	0.16, 0.36, 0.8, 1.8 and 4.0 μg/L, 24 h	The mortality rate was 80% at 0.16 and 0.36 μg/L groups, and 100% in the 0.8, 1.8 and 4.0 μg/L groups. Symptoms developed within 40 minutes in the 4.0 μg/L group, and all died within less than 2 hours.	[45]
			0.02, 0.04, 0.06, 0.1 and 0.2 μg/L, 24 h	LC_{50} = 95 ng/L.The mortality rate was 100% at 0.2 μg/L, 67% at 0.1 μg/L, and no death occurred at 0.02, 0.04 and 0.06 μg/L.	
	TWP leachate	adult coho salmon (*Oncorhynchus kisutch*)	100, 320 and 1000 mg/L, 24 h	The mortality rate was 25-50% at 100mg/L and 100% at 320, 1000 mg/L groups. Most deaths occur within 6 hours. A significant increase in hematocrit and decrease in plasma Na and pH. Leukocrit, pCO_2, plasma chloride, and glucose were not significantly affected.	[46]
	TWP leachate	adult chum salmon (*Oncorhynchus keta*)	100, 320 and 1000 mg/L, 24 h	No effects.	
	6PPD-quinone	Juvenile Chinook (*Oncorhynchus tshawytscha*) (~3	5716, 10288, 8519, 33333 and 60000 ng/L, 24 h, 13.6°C	LC_{50} >67,306.8 ng/L. LC_5=12,613.8 ng/L. LC_{10}=20,858.9 ng/L. LC_{25}=43,698.7 ng/L. An average survival rate of 61.4% is in the highest treatment group (67,306.8 ng/L). Fishes exhibited typical	[47]

	weeks)		6PPD-quinone symptomology (e.g. gasping, loss of equilibrium, erratic swimming), and fish that were symptomatic generally died from toxic injury.	
	juvenile coho (*Oncorhynchus kisutch*) (~3 weeks)	11.9, 21.4, 38.6, 69.4 and 125 ng/L, 24 h, 13.8℃	LC_{50}=41.0 ng/L. An average of 7.1% survival is in the highest treatment group (104.7 ng/L). Coho exhibited symptoms and mortality occurred during the fourth hour of exposure at the highest test concentration (104.7 ng/L). Fishes exhibited typical 6PPD-quinone symptomology (e.g. gasping, loss of equilibrium, erratic swimming), and fish that were symptomatic generally died from toxic injury.	
diluted urban roadway runoff	juvenile coho (*Oncorhynchus kisutch*) (>1 year)	1, 2.2, 5.0, 11.2 and 25% of untreated roadway runoff in clean water for 24 h, then to 48 h clean water, 10~12.1℃	5% or over 5% were generally lethal.	[21]
untreated roadway runoff	juvenile coho (*Oncorhynchus kisutch*) (<1 year)	3 untreated roadway runoffs for 24 h, then to 48 h clean water, 5.0~10.3℃	Cumulative mortality rates ranging from 92~100% in all runoffs. Fishes began dying soon during exposure (2-4 h), with near-maximal cumulative mortality within 8 h. Died rapidly(<4h) and had visible distress (disorientation, loss of equilibrium).	
	juvenile coho (*Oncorhynchus kisutch*) (>1year)		Cumulative mortality rates ranging from 92~100% in all runoffs. Fishes began dying soon during exposure (2-4 h), with near-maximal cumulative mortality within 8 h.	
	juvenile steelhead (*Oncorhynchus mykiss*) (>1year)		Cumulative mortality rates ranging from 4-42%. Fish generally dying toward the end of the 24 h exposure or after subsequent transfer to clean water, with surface swimming and gaping, loss of equilibrium.	
	subyearling Chinook		Cumulative mortality rates ranging from 0~13%. Deaths	

	Salmonids (*Oncorhynchus tshawytscha*) (<1 year)		occurred within 1-2 days, with surface swimming and gaping, loss of equilibrium.	
	juvenile Congeneric Sockeye (*Oncorhynchus nerka*) <1 year		100% survived.	
rac-6PPD-quinone (racemate)	*Oncorhynchus mykiss*	1.75-2.56 mg/L, 96 h, 16°C	LC_{50}=2.26 μg/L.	[46]
R-6PPD-quinone (R-enantiomer)		2.05-5.00 mg/L, 96 h, 16°C	LC_{50}=4.31 μg/L.	
S-6PPD-quinone (S-enantiomer)		1.31-1.92 mg/L, 96 h, 16°C	LC_{50}=1.66 μg/L.	
6PPD-quinone	Rainbow Trout (*Oncorhynchus mykiss*) (< 2 years)	0.15, 0.75, 1.5, 3 and 6 μg/L, 96 h, 12°C	24 hLC_{50}=1.96 μg/L, 72-96 hLC_{50}=1.00 μg/L. The first signs of morbidity did not manifest until 7 h after commencing exposures and maximum mortalities occurred at 60 h; The maximum mortality was observed at 60 h in 1.5 μg/L treatment group. At 3 and 6 μg/L groups, the mortality rate was 100% after 24 h, which was accompanied by increased respiration, wheezing, spiraling behavior, loss of balance, and significantly increased blood glucose concentration. Behaviors in deaths: hovering close to the water surface, accelerated opercular movements, gasping, and spiraling motion. A significant increase in blood glucose concentrations observed at 2.78 μg/L indicates that 6PPD-	[49]

			quinone impacted energy metabolism.	
	Brook Trout (*Slavelinus fontinslis*) (<1 year)	0.1, 0.5, 1, 2 and 4 μg/L, 24 h, 10℃	24 hLC_{50}=0.59 μg/L. The mortality rate of 4 μg/L treatment group at 3 hours was 100%. Behaviors in deaths: hovering close to the water surface, accelerated opercular movements, gasping, and spiraling motion. A significant increase in blood glucose concentrations observed at 0.72−2.21 μg/L indicates that 6PPD-quinone impacted energy metabolism. Hematocrit of fish exposed to 0.72−4.35 μg/L 6PPD-quinone significantly increased from an average of 42% in the control group to 68% at 4.35 μg/L.	
	Arctic char (*Slavelinus alpinus*) (< 3 years)	20 μg/L, 96 h, 12℃	No mortalities were observed at measured concentration of 14.2 μg/L.	
6PPD-quinone	White-spotted char (*Salvelinus lencomaenis pluvius*) (<1 year)	1 and 10 μg/L, 96 h, 14℃	The mortality of 1 μg/L treatment group was 50%. The mortality of 10 μg/L treatment group was 100%.	[40]
		0, 0.25, 0.5, 1, 2 and 4 μg/L, 96 h, 14℃	24 h LC_{50}=0.51 μg/L. All deaths occurred within 24 hours of exposure; All deaths were observed within 4 h at 3.5 μg/L. Abnormal swimming behavior (including hovering close to the water surface, erratic swimming, and tumbling), hovering near the surface, swimming and rolling erratically. White mold-like stuff was found at concentrations and times where abnormal behaviors were observed.	
	Southern Asian Dolly Varden (*Salvelinus curilus*) <1 year	1 and 10 μg/L, 96 h, 14℃	Non-lethal toxicity. No symptoms of abnormal behavior.	

		Landlocked masu salmon (*Oncorhynchus masou masou*) (<1 year)	1 and 10 μg/L, 96 h, 14℃	Non-lethal toxicity; No symptoms of abnormal behavior.	
	6PPD-quinone	Atlantic salmon (*salmo trutta*) (<1 year)	0.095, 0.19, 0.38, 0.76, 1.52, 3.04, 6.08, and 12.16 μg/L, 48h, 4.9℃	No mortalities or substantial changes in behavior.	[50]
		Brown throut alevins (*salmo salar*) (<1 year)	0.095, 0.19, 0.38, 0.76, 1.52, 3.04, 6.08, and 12.16 μg/L, 48h, 4.9℃	No mortalities or substantial changes in behavior.	
Zebrafish	6PPD	Zebrafish embryo (*Danio rerio*) (16-cell stage eggs)	895 and 792 μg/L (maximum water solubility), 96 h, 26℃	Incubation rate delay (45% after 72 h). The body length of fish decreases. 25% of zebrafish have curvature of the spine.	[51]
	6PPD-quinone		70 and 71 μg/L (maximum water solubility), 96 h, 26℃	No visible symptoms of abnormal behavior.	
	6PPD	adult wild-type zebrafish (four months old)	50, 500 and 1000 μg/L, 12 h, 26℃	The movement velocity of zebrafish decreased with the increase of exposure concentration, and the movement distance decreased obviously and the body mendering increased obviously. Zebrafish cannot move forward normally in the first 3 hours; Surface retention time decreased by 91% and anxiety level increased significantly. A slight increase in dopamine levels, a significant increase in GABA levels, a significant decrease in acetylcholine levels, may have Huntington's Dance disease. Abnormal swimming posture.	[52]
	6PPD-quinone		1000 μg/L, 12 h, 26℃	Reduced exercise rate, significantly increased mendering,	

			increased dopamine and GABA levels, decreased acetylcholine levels, and possibly Huntington's Dance disease.	
6PPD	zebrafish larvae (116 hpf)	0-1500 μg/L, 96 h	24 hLC_{50}=1384.9 μg/L, 48hLC_{50}=816.9 μg/L, 72 hLC_{50}=609.4 μg/L, 96 hLC_{50}=442.6 μg/L.	[53]
6PPD-quinone		0-1000 μg/L, 96 h	24 hLC_{50}=308.6 μg/L, 48hLC_{50}=224.6 μg/L, 72 hLC_{50}=171.6 μg/L, 96 hLC_{50}=132.9 μg/L.	
6PPD		0, 1.0, 10.0 and 25.0 μg/L, 96 h	The hatching rate, survival rate and body length of zebrafish were not significantly affected. Increased oxygen consumption. When exposure concentration was 25.0 μg/L, the yolk absorption, bladder, heartbeat and travel distance of zebrafish were decreased significantly. When the exposure concentration was 10.0 or 25.0 μg/L, the size of zebrafish eyes decreased significantly.	
6PPD-quinone		0, 1.0, 10.0 and 25.0 μg/L, 96 h	Increased oxygen consumption. When exposure concentration was 10.0 or 25.0 μg/L, fish intestinal tract became red, heart rate decreased significantly, and moving distance decreased significantly. When the exposure concentration was 25.0 μg/L, the body of zebrafish was deformed and the eye size was significantly reduced.	
6PPD	zebrafish embryos/larvae (2hpf)	0, 0.0022, 0.022 and 0.22 mg/L, 120h	96 hLC50=2.2 mg/L. Incubation rate decreased significantly after 48 h exposure. After 96 h of exposure, movement decreased, tail curvature and spinal deformity appeared. 120h after exposure, body length decreased and thyroid hormone concentration increased; When exposed to 0.22 mg/L high concentration, gene expression level, antioxidant enzyme activity, hatching rate and autonomic movement decreased by	[54]

				78.9% and 56.25% respectively.	
	6PPD	zebrafish larvae (2-96 hpf)	250-1750 μg/L, 7 days<br>10, 100, 300, 500 and 700 μg/L	LC_{50}=737 μg/L (12 h).<br>6PPD had potential cardiotoxicity in zebrafish larvae. Developmental disorders, including the malabsorption of the yolk sac and heart malformation. The genes associated with cardiac functions, including the calcium signal pathway and myocardial contraction, were enriched in the KEGG (Kyoto Encyclopedia of Genes and Genomes) pathway enrichment analysis. The expression of the genes essential to the development of the cardiac system decreased significantly, and the cardiac system of zebrafish larvae was deformed as it developed.	[56]
Adrianichthyidae	6PPD	*Oryzias latipes* (41 days old)	286 and 382 μg/L (maximum water solubility), 96 h, 24.5°C	The mortality rate was 80% at 107 μg/L and abnormal swimming behaviors (e.g., surfacing or swimming near the bottom) occurred within 1h.	[51]
	6PPD-quinone		40 and 58 μg/L (maximum water solubility), 96 h, 24.5°C	No acute death and no behavioral symptoms.	
Amphipoda	6PPD	*Hyalella azteca* (3–5-days-old)	~ 680 μg/L (maximum water solubility), 48h, 23.5°C	The mortality rate is 100% at 138 μg/L.	
	6PPD-quinone		~ 60 μg/L (maximum water solubility), 48h, 23.5°C	No acute death.	
Pulicidae	6PPD	*Daphnia magna* (< 24 h old)	750 and 990 μg/L (maximum water solubility), 96 h, 21.5°C	The mortality rate is 100% at 286 μg/L.	
	6PPD-quinone		51 and 94 μg/L (maximum water solubility), 96 h, 21.5°C	No acute death.	

Cyprinidae	rac-6PPD	*Gobiocypris rarus*	112-233 mg/L, 96 h, 25℃	LC_{50}=162 μg/L.	[48]
	R-6PPD		194-402 mg/L, 96 h, 25℃	LC_{50}=201 μg/L. The toxicities of S-6PPD and R-6PPD were almost the same. The TU_{rac} was 0.806, so the toxic interaction was concentration addition.	
	S-6PPD		194-402 mg/L, 96 h, 25℃	LC_{50}=201 μg/L.	
	rac-6PPD-quinone<br>R-6PPD-quinone<br>S-6PPD-quinone			No fish died in rac-6PPD-quinone, R-6PPD-quinone and S-6PPD-quinone test solutions at 500 μg/L.	
Acipenseridae	6PPD-quinone	white sturgeon (*Acipenser transmontanus*)	20 μg/L, 96 h, 12℃	No mortalities were observed at measured concentration of 12.7 μg/L. Estimated LC_{50}>12,700 ng/L.	[49]
Brachionidae	6PPD	*Brachionus calyciflorus*	0, 125, 250, 500 and 1000 μg/L, 12days	Population growth is affected, and growth and reproduction rates are reduced.	[55]
	6PPD-quinone		1000 μg/L, 12days	It has little effect on population growth.	
Nematode	6PPD-quinone	*Caenorhabditis elegans* (L1-larve)	0.1 ~ 100 μg/L, approximately 4.5 days	100 μg/L group can cause the lethality; the intestinal permeability was significantly enhanced by 1–10 μg/L as indicated by erioglaucine disodium staining; the expression of intestinal fatty acid transporter ACS-22 governing functional state of intestinal barrier was decreased by exposure to 1–10 μg/L; intestinal reactive oxygen species (ROS) production was induced by 0.1–10 μg/L and lipofuscin accumulation reflected by intestinal autofluorescence was activated by 1–10 μg/L. Accompanied with activation of intestinal oxidative stress, expressions of some anti-oxidation related genes (ctl-2, sod-2, sod-3, a n d sod-4) were significantly increased by 0.1–10 μg/L; Intestinal RNAi of acs-	[56]

				22 strengthened the susceptibility of nematodes to intestinal toxicity.	
Earthworm	TRWPs	*Eisenia fetida*	0.05% and 5% CMTT, contaminated soil (100 g/L), 4 weeks	Tire-related chemicals exhibit a low bioaccumulation potential; no mortality or impact on growth in the adult worm population was observed in all treatments tested; individual weight loss.	[57]
Plant	6PPD	lettuce	1 mg/L, 14 days	6PPD can be detected in roots and leaves. 6PPD is readily taken up by lettuce with measured maximum leaf concentrations between ~0.78 μg/g (7th day).	[42]
	6PPD-quinone		1 mg/L, 14 days	6PPD-quinone can be detected in roots and leaves and most retained in roots.	
Mammal	6PPD<br>6PPD-quinone	male mice	10, 30 and 100 mg/kg, 22℃, 6 weeks<br>10, 30 and 100 mg/kg, 22℃, 6 weeks	Body weight did not change significantly, but liver weight increased significantly. Hepatotoxicity: abnormal lipid metabolism, liver cell injury, liver fibrosis, liver bile production and excretion. The 6PPD and 6PPD-quinone accumulated in the liver in a dose-dependent manner, and the levels of 6PPD and 6PPD-quinone even reached as high as 681.9±65.0 and 1194.7±515.9 ng/g liver weight respectively when exposed to 100 mg/kg 6PPD and 6PPD-quinone.	[44]

Reference

1. Wang, W.; Cao, G.; Zhang, J.; Wu, P.; Chen, Y.; Chen, Z.; Qi, Z.; Li, R.; Dong, C.; Cai, Z. Beyond substituted *p*-phenylenediamine antioxidants: prevalence of their quinone derivatives in $PM_{2.5}$. *Environ. Sci. Technol.* **2022**, *56*, 10629–10637.

2. Wang, W.; Cao, G.; Zhang, J.; Chen, Z.; Dong, C.; Chen, J.; Cai, Z. *p*-Phenylenediamine-derived quinones as new contributors to the oxidative potential of fine particulate matter. *Environ. Sci. Technol. Lett.* **2022**, *9*, 712–717.

3. Zhang, Y.; Xu, C.; Zhang, W.; Qi, Z.; Song, Y.; Zhu, L.; Dong, C.; Chen, J.; Cai, Z. *p*-Phenylenediamine antioxidants in $PM_{2.5}$: The underestimated urban air pollutants. *Environ. Sci. Technol.* **2022**, *56*, 6914–6921.

4. Cao, G.; Wang, W.; Zhang, J.; Wu, P.; Zhao, X.; Yang, Z.; Hu, D.; Cai, Z. New evidence of rubber-derived quinones in water, air, and soil. *Environ. Sci. Technol.* **2022**, *56*, 4142–4150.

5. Zhang, Y.J.; Xu, T.T.; Ye, D.M.; Lin, Z.Z.; Wang, F.; Guo, Y. Widespread *N*-(1,3-Dimethylbutyl)-*N′*-Phenyl-*p*-Phenylenediamine quinone in size-fractioned atmospheric particles and dust of different indoor environments. *Environ. Sci. Technol. Lett.* **2022**, *9*, 420–425.

6. Johannessen, C.; Saini, A.; Zhang, X.; Harner, T. Air monitoring of tire-derived chemicals in global megacities using passive samplers. *Environ. Pollut.* **2022**, *314*, 120206.

7. Huang, W.; Shi, Y.; Huang, J.; Deng, C.; Tang, S.; Liu, X.; Chen, D. Occurrence of substituted *p*-phenylenediamine antioxidants in dusts. *Environ. Sci. Technol. Lett.* **2021**, *8*, 381–385.

8. Deng, C.; Huang, J.; Qi, Y.; Chen, D.; Huang, W. Distribution patterns of rubber tire-related chemicals with particle size in road and indoor parking lot dust. *Sci. Total Environ.* **2022**, *844*, 157144.

9. Liang, B.; Li, J.; Du, B.; Pan, Z.; Liu, L.Y.; Zeng, L. E-waste recycling emits large quantities of emerging aromatic amines and organophosphites: A poorly recognized source for another two classes of synthetic antioxidants. *Environ. Sci. Technol. Lett.* **2022**, *9*, 625–631.

10. Zhang, Z.; Xu, X.; Qian, Z.; Zhong, Q.; Wang, Q.; Hylkema, M.N.; Snieder, H.; Huo, X. Association between 6PPD-quinone exposure and bmi, influenza, and diarrhea in children. *Environ. Res.* **2024**, *247*, 118201.

11. Zhang, Z.; Dai, C.; Chen, S.; Hu, H.; Kang, R.; Xu, X.; Huo, X. Spatiotemporal variation of 6PPD and 6PPDQ in dust and soil from e-waste recycling areas. *Sci. Total Environ.* **2024**, *923*, 171495.

12. Liu, R.; Li, Y.; Lin, Y.; Ruan, T.; Jiang, G. Emerging aromatic secondary amine contaminants and related derivatives in various dust matrices in China. *Ecotox. Environ. Safe.* **2019**, *170*, 657–663.

13. Zhang, Y.; Geng, N.; Yan, L.; Wang, L.; Zhang, H.; Chen, J. Distribution characteristics and human exposure assessment of *p*-Phenylenediamine (PPD) antioxidants and their oxidation products in road dust and nearby topsoil. *Asian J. Ecotox.* **2023**, *18*, 253–264.(In Chinese)
14. Zhang, Y.; Li, J.N.; Wang, J.X.; Hu, J.; Sun, J.L.; Li, Y.F.; Li, W.L.; Tang, Z.H.; Zhang, Z.F. Aniline antioxidants in road dust, parking lot dust, and green-belt soil in harbin, a megacity in China: Occurrence, profile, and seasonal variation. *J. Hazard. Mater.* **2024**, *469*, 134001.
15. Zhu, J.; Guo, R.; Jiang, S.; Wu, P.; Jin, H. Occurrence of *p*-phenylenediamine antioxidants (PPDs) and PPDs-derived quinones in indoor dust. *Sci. Total Environ.* **2024**, *912*, 169325.
16. Zhang, Y.; Yan, L.; Wang, L.; Zhang, H.; Chen, J.; Geng, N. A nation-wide study for the occurrence of PPD antioxidants and 6PPD-quinone in road dusts of China. *Sci. Total Environ.* **2024**, *922*, 171393.
17. Hiki, K.; Yamamoto, H. Concentration and leachability of N-(1,3-dimethylbutyl)-N′-phenyl-p-phenylenediamine (6PPD) and its quinone transformation product (6PPD-Q) in road dust collected in Tokyo, Japan. *Environ. Pollut.* **2022**, *302*, 119082.
18. Klöckner, P.; Seiwert, B.; Weyrauch, S.; Escher, B.I.; Reemtsma, T.; Wagner, S. Comprehensive characterization of tire and road wear particles in highway tunnel road dust by use of size and density fractionation. *Chemosphere* **2021**, *279*, 130530.
19. Tian, Z.; Zhao, H.; Peter, K.T.; Gonzalez, M.; Wetzel, J.; Wu, C.; Hu, X.; Prat, J.; Mudrock, E.; Hettinger, R.; et al. A ubiquitous tire rubber–derived chemical induces acute mortality in coho salmon. *Science* **2021**, *371*, 185–189.
20. French, B.F.; Baldwin, D.H.; Cameron, J.; Prat, J.; King, K.; Davis, J.W.; McIntyre, J.K.; Scholz, N.L. Urban roadway runoff is lethal to juvenile coho, steelhead, and chinook salmonids, but not congeneric sockeye. Environ. Sci. Technol. Lett. 2022, 9, 733–738.
21. Cao, G.; Wang, W.; Zhang, J.; Wu, P.; Qiao, H.; Li, H.; Huang, G.; Yang, Z.; Cai, Z. Occurrence and fate of substituted p-phenylenediamine-derived quinones in Hong Kong wastewater treatment plants. *Environ. Sci. Technol.* **2023**, *57*, 15635–15643.
22. Challis, J.K.; Popick, H.; Prajapati, S.; Harder, P.; Giesy, J.P.; McPhedran, K.; Brinkmann, M. Occurrences of tire rubber-derived contaminants in cold-climate urban runoff. *Environ. Sci. Technol. Lett.* **2021**, *8*, 961–967.
23. Johannessen, C.; Helm, P.; Metcalfe, C.D. Detection of selected tire wear compounds in urban receiving waters. *Environ. Pollut.* **2021**, *287*, 117659.
24. Johannessen, C.; Helm, P.; Lashuk, B.; Yargeau, V.; Metcalfe, C.D. The tire wear compounds 6PPD-quinone and 1,3-diphenylguanidine in an urban watershed. *Arch. Environ. Contam. Toxicol.* **2022**, *82*, 171–179.
25. Rauert, C.; Charlton, N.; Okoffo, E.D.; Stanton, R.S.; Agua, A.R.; Pirrung, M.C.; Thomas, K.V. Concentrations of tire additive chemicals and

tire road wear particles in an Australian urban tributary. *Environ. Sci. Technol.* **2022**, *56*, 2421–2431.
26. Rauert, C.; Vardy, S.; Daniell, B.; Charlton, N.; Thomas, K.V. Tyre additive chemicals, tyre road wear particles and high production polymers in surface water at 5 urban centres in Queensland, Australia. *Sci. Total Environ.* **2022**, *852*, 158468.
27. Monaghan, J.; Jaeger, A.; Agua, A.R.; Stanton, R.S.; Pirrung, M.; Gill, C.G.; Krogh, E.T. A direct mass spectrometry method for the rapid analysis of ubiquitous tire-derived toxin N-(1,3-dimethylbutyl)-N′-phenyl-p-phenylenediamine quinone (6-PPDQ). *Environ. Sci. Technol. Lett.* **2021**, *8*, 1051–1056.
28. Seiwert, B.; Nihemaiti, M.; Troussier, M.; Weyrauch, S.; Reemtsma, T. Abiotic oxidative transformation of 6-PPD and 6-PPD quinone from tires and occurrence of their products in snow from urban roads and in municipal wastewater. *Water Res.* **2022**, *212*, 118122.
29. Zhang, R.; Zhao, S.; Liu, X.; Thomes, M.W.; Bong, C.W.; N. D. Samaraweera, D.; Priyadarshana, T.; Zhong, G.; Li, J.; Zhang, G. Fates of benzotriazoles, benzothiazoles, and p-phenylenediamines in wastewater treatment plants in Malaysia and Sri Lanka. *ACS EST Water* **2023**, *3*, 1630–1640.
30. Zhang, R.; Zhao, S.; Liu, X.; Tian, L.; Mo, Y.; Yi, X.; Liu, S.; Liu, J.; Li, J.; Zhang, G. Aquatic environmental fates and risks of benzotriazoles, benzothiazoles, and *p*-phenylenediamines in a catchment providing water to a megacity of China. *Environ. Res.* **2023**, *216*, 114721.
31. Zhu, J.; Guo, R.; Ren, F.; Jiang, S.; Jin, H. Occurrence and partitioning of *p*-phenylenediamine antioxidants and their quinone derivatives in water and sediment. *Sci. Total Environ.* **2024**, *914*, 170046.
32. Zhang, H.Y.; Huang, Z.; Liu, Y.H.; Hu, L.X.; He, L.Y.; Liu, Y.S.; Zhao, J.L.; Ying, G.G. Occurrence and risks of 23 tire additives and their transformation products in an urban water system. *Environ. Int.* **2023**, *171*, 107715.
33. Zeng, L.; Li, Y.; Sun, Y.; Liu, L.Y.; Shen, M.; Du, B. Widespread occurrence and transport of *p*-phenylenediamines and their quinones in sediments across urban rivers, estuaries, coasts, and deep-sea regions. *Environ. Sci. Technol.* **2023**, *57*, 2393–2403.
34. Zhang, X.; Peng, Z.; Hou, S.; Sun, Q.; Yuan, H.; Yin, D.; Zhang, W.; Zhang, Y.; Tang, J.; Zhang, S.; et al. Ubiquitous occurrence of p-phenylenediamine (PPD) antioxidants and PPD-quinones in fresh atmospheric snow and their amplification effects on associated aqueous contamination. *J. Hazard. Mater.* **2024**, *465*, 133409.
35. Schneider, K.; De Hoogd, M.; Madsen, M.P.; Haxaire, P.; Bierwisch, A.; Kaiser, E. ERASSTRI - European risk assessment study on synthetic turf rubber infill – Part 1: Analysis of infill samples. *Sci. Total Environ.* **2020**, *718*, 137174.
36. Zhao, H.N.; Hu, X.; Gonzalez, M.; Craig, A.R.; Grant, C.H.; Matthew, F.F.; Carter, J.M.; Micheal, C.D.; Kelly, E.K.; Tian, Z.; et al. Screening

p-phenylenediamine antioxidants, their transformation products, and industrial chemical additives in crumb rubber and elastomeric consumer products. *Environ. Sci. Technol.* **2023**, *57*, 2779–2791.
37. Du, B.; Liang, B.; Li, Y.; Shen, M.; Liu, L.Y.; Zeng, L. First report on the occurrence of N-(1,3-dimethylbutyl)-N′-phenyl-p-Phenylenediamine (6PPD) and 6PPD-quinone as pervasive pollutants in human urine from south China. *Environ. Sci. Technol. Lett.* **2022**, *9*, 1056–1062.
38. Mao, W.; Jin, H.; Guo, R.; Chen, P.; Zhong, S.; Wu, X. Occurrence of p-phenylenediamine antioxidants in human urine. *Sci. Total Environ.* **2024**, *914*, 170045.
39. Song, S.; Gao, Y.; Feng, S.; Cheng, Z.; Huang, H.; Xue, J.; Zhang, T.; Sun, H. Widespread occurrence of two typical N, N'-substituted p-phenylenediamines and their quinones in humans: Association with oxidative stress and liver damage. *J. Hazard. Mater.* **2024**, *468*, 133835.
40. Hiki, K.; Yamamoto, H. The tire-derived chemical 6PPD-quinone is lethally toxic to the white-spotted char *Salvelinus Leucomaenis Pluvius* but not to two other salmonid species. *Environ. Sci. Technol. Lett.* **2022**, *9*, 1050–1055.
41. Fang, C.; Fang, L.; Di, S.; Yu, Y.; Wang, X.; Wang, C.; Jin, Y. Characterization of N-(1,3-dimethylbutyl)-N′-phenyl-*p*-phenylenediamine (6PPD)-induced cardiotoxicity in larval zebrafish (*Danio Rerio*). *Sci. Total Environ.* **2023**, *882*, 163595.
42. Castan, S.; Sherman, A.; Peng, R.; Zumstein, M.T.; Wanek, W.; Hüffer, T.; Hofmann, T. Uptake, metabolism, and accumulation of tire wear particle-derived compounds in lettuce. *Environ. Sci. Technol.* **2023**, *57*, 168–178.
43. Ji, J.; Li, C.; Zhang, B.; Wu, W.; Wang, J.; Zhu, J.; Liu, D.; Gao, R.; Ma, Y.; Pang, S.; et al. Exploration of emerging environmental pollutants 6PPD and 6PPDQ in honey and fish samples. *Food Chem.* **2022**, *396*, 133640.
44. Fang, L.; Fang, C.; Di, S.; Yu, Y.; Wang, C.; Wang, X.; Jin, Y. Oral exposure to tire rubber-derived contaminant 6PPD and 6PPD-quinone induce hepatotoxicity in mice. *Sci. Total Environ.* **2023**, *869*, 161836.
45. Tian, Z.; Gonzalez, M.; Rideout, C.A.; Zhao, H.N.; Hu, X.; Wetzel, J.; Mudrock, E.; James, C.A.; McIntyre, J.K.; Kolodziej, E.P. 6PPD-Quinone: Revised toxicity assessment and quantification with a commercial standard. *Environ. Sci. Technol. Lett.* **2022**, *9*, 140–146.
46. McIntyre, J.K.; Prat, J.; Cameron, J.; Wetzel, J.; Mudrock, E.; Peter, K.T.; Tian, Z.; Mackenzie, C.; Lundin, J.; Stark, J.D.; et al. Treading water: Tire wear particle leachate recreates an urban runoff mortality syndrome in coho but not chum salmon. *Environ. Sci. Technol.* **2021**, *55*, 11767–11774.
47. Lo, B.P.; Marlatt, V.L.; Liao, X.; Reger, S.; Gallilee, C.; Ross, A.R.S.; Brown, T.M. Acute toxicity of 6PPD-quinone to early life stage juvenile chinook (*Oncorhynchus Tshawytscha*) and coho (*Oncorhynchus Kisutch*) salmon. *Environ. Toxicol. Chem.* **2023**, *42*, 815–822.

48. Di, S.; Liu, Z.; Zhao, H.; Li, Y.; Qi, P.; Wang, Z.; Xu, H.; Jin, Y.; Wang, X. Chiral perspective evaluations: Enantioselective hydrolysis of 6PPD and 6PPD-quinone in water and enantioselective toxicity to *Gobiocypris Rarus* and *Oncorhynchus Mykiss*. *Environ. Int.* **2022**, *166*, 107374.
49. Brinkmann, M.; Montgomery, D.; Selinger, S.; Miller, J.G.P.; Stock, E.; Alcaraz, A.J.; Challis, J.K.; Weber, L.; Janz, D.; Hecker, M.; et al. Acute toxicity of the tire rubber-derived chemical 6PPD-quinone to four fishes of commercial, cultural, and ecological importance. *Environ. Sci. Technol. Lett.* **2022**, *9*, 333–338.
50. Foldvik, A.; Kryuchkov, F.; Sandodden, R.; Uhlig, S. Acute toxicity testing of the tire rubber–derived chemical 6PPD-quinone on atlantic salmon (*Salmo Salar*) and brown trout (*Salmo Trutta*). *Enviro. Toxic and Chemistry* **2022**, *41*, 3041–3045.
51. Hiki, K.; Asahina, K.; Kato, K.; Yamagishi, T.; Omagari, R.; Iwasaki, Y.; Watanabe, H.; Yamamoto, H. Acute toxicity of a tire rubber-derived chemical, 6PPD-quinone, to freshwater fish and crustacean species. *Environ. Sci. Technol. Lett.* **2021**, *8*, 779–784.
52. Ji, J.; Huang, J.; Cao, N.; Hao, X.; Wu, Y.; Ma, Y.; An, D.; Pang, S.; Li, X. Multiview behavior and neurotransmitter analysis of zebrafish *Dyskinesia* induced by 6PPD and its metabolites. *Sci. Total Environ.* **2022**, *838*, 156013.
53. Varshney, S.; Gora, A.H.; Siriyappagouder, P.; Kiron, V.; Olsvik, P.A. Toxicological effects of 6PPD and 6PPD-quinone in zebrafish larvae. *J. Hazard. Mater.* **2022**, *424*, 127623.
54. Peng, W.; Liu, C.; Chen, D.; Duan, X.; Zhong, L. Exposure to N-(1,3-dimethylbutyl)-N′-phenyl-p-phenylenediamine (6PPD) affects the growth and development of zebrafish embryos/larvae. *Ecotox. Environ. Safe.* **2022**, *232*, 113221.
55. Klauschies, T.; Isanta.N., J. The joint effects of salt and 6PPD contamination on a freshwater *Herbivore*. *Sci. Total Environ.* **2022**, *829*, 154675.
56. Hua, X.; Feng, X.; Liang, G.; Chao, J.; Wang, D. Long-term exposure to tire-derived 6-PPD quinone causes intestinal toxicity by affecting functional state of intestinal barrier in *Caenorhabditis Elegans*. *Sci. Total Environ.* **2023**, *861*, 160591.
57. Domínguez, A.; Brown, G.G.; Sautter, K.D.; Ribas De Oliveira, C.M.; De Vasconcelos, E.C.; Niva, C.C.; Bartz, M.L.C.; Bedano, J.C. Toxicity of AMPA to the earthworm *Eisenia Andrei Bouché*, 1972 in Tropical Artificial Soil. *Sci. Rep.* **2016**, *6*, 19731.
